# Correction in Active Cases Data of COVID-19 for the US States by Analytical Study

**DOI:** 10.1017/dmp.2021.130

**Published:** 2021-04-30

**Authors:** Ravi Solanki, Anubhav Varshney, Raveesh Gourishetty, Saniya Minase, Namitha Sivadas, Ashutosh Mahajan

**Affiliations:** 1 Centre for VLSI and Nanotechnology, Visvesvaraya National Institute of Technology, Nagpur, India; 2 Department of Electrical Engineering, Swami Vivekanand College of Engineering, Indore, India; 3 Department of Electrical Engineering, Indian Institute of Technology Bombay, Mumbai, India; 4 Department of Electrical and Electronics Engineering, Birla Institute of Technology and Science Pilani, Goa, India; 5 Centre for Nanotechnology Research, Vellore Institute of Technology, Vellore, India

**Keywords:** COVID-19, epidemiology, mathematical model

## Abstract

The total coronavirus disease (COVID-19) cases caused by the severe acute respiratory syndrome coronavirus 2 (SARS-CoV-2) infection have reached 139 million worldwide and nearing 3 million deaths on April 16, 2021. The availability of accurate data is crucial as it makes it possible to analyze correctly the infection trends and make better forecasts. The reported recovered cases for many US states are surprisingly low. This could be due to difficulties in keeping track of recoveries, which resulted in higher numbers for the reported active cases than the actual numbers on the ground. In this work, based on the typical range of recovery rate for COVID-19, we estimate the active data from the total cases and death cases and bring out a correction for the data for all the US states reported on Worldometer.

## Introduction

The availability of accurate data of an epidemic is important as the data provide key insights on the disease spread and enable the authorities to take a decision on control measures. Worldometer is one of the very popular sources of the global coronavirus disease (COVID-19) data, and it is also trusted and used by many government bodies and agencies.^1^ The available data for the COVID-19 cases can be used for the prediction and analysis of hospitalization and meeting the demands of health care facilities and setting up the critical care systems for the patients. The active cases represent the number of infected people, whether symptomatic or asymptomatic, detected through self-reporting or testing. This number is important for public health authorities to estimate the current status of the disease spread and can be calculated by subtracting death and recovered cases from the total confirmed cases.

## Method

A compartmental predictive mathematical model, SIPHERD, for COVID-19 dynamics was used where the recovery rate is a model parameter and is fixed by optimizing the model with the actual data.^2,3^ The data for the total death and active cases for 364 days from March 4, 2020, were taken from Worldometer^1^ and data found were close to total cases and death data from one study.^4^ After running the SIPHERD model for the United States, as reported in another study,^2^ the recovery rate of the active category was found to be 0.015 (corresponding to 66 days of mean recovery time), which is very low compared with other countries like Germany and India,^2,3^ where the recovery rate was 0.065. The low recovery rate in the United States may be attributed to either incorrect reporting of active cases^5^ or the testing of only serious cases and a longer recovery time in hospitals compared with quarantined with mild symptoms. Second, keeping the record of recoveries is difficult because some of the infected people are asked to quarantine, whereas only critical patients are hospitalized. Sometimes, the reporting of those recoveries is not accurate or incomplete. This has led to inconsistent data for active cases.

The number of mild cases is reported to be 81% in a Chinese study.^6^ COVID-19 data reported from 49 states, the District of Columbia, and 3 US territories to the Centers for Disease Control and Prevention from February 12–March 16 show that 20.7 reported cases were severe and patients were hospitalized.^7^ COVID-NET regions show this number to be 21.4% till April 4^8^ and Institute for Health Metrics and Evaluation data from March 5–April 4 show this number to be 20.3%.^9^ According to the World Health Organization, the recovery time for mild cases is 2 weeks and 3–6 weeks for severe cases. Considering 80% of mild cases, the recovery rate cannot be as low as it appears in the data for the states listed in column 2 of Table 1.

**Table 1.** The US states active cases data status

Correct Data	Incorrect Data	Partially Correct Data
Texas	Maryland	Nebraska
Tennessee	Virginia	Indiana
Louisiana	Maine	California
Arizona	Hawaii	Oregon
North Carolina	Kentucky	Florida
Illinois	Minnesota	Idaho
Massachusetts	Rhode Island	New Mexico
Pennsylvania	South Carolina	Louisiana
Ohio	Washington	Alabama
Arkansas		Michigan
Connecticut		Alaska
Delaware		Colorado
Iowa		Missouri
Kansas		New Jersey
Mississippi		Georgia
Montana		New York
North Dakota		District of Columbia
New Hampshire		
Oklahoma		
South Dakota		
Utah		
Vermont		
West Virginia		
Wisconsin		
Wyoming		

The correct estimation of the active cases can be done by subtracting the death and recovery cases, with the appropriate recovery rate, from the total cases. The Worldometer data for total cases and death cases are assumed to be true as the testing for positive results and recording of diseased cases are done more stringently as compared to recovery counting. The active cases can be obtained by using the following differential equation:
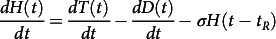



where, 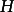 ,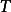, and 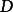 are the active, total, and death cases, respectively. The recovery from the “infected” category is defined by the 2 parameters: delay in recovery 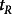 and the recovery rate 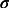. As these values are dependent upon the immune system of the community and the hospital facilities, it should not vary much within the United States. We have taken 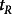 as 10 days and 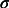 as 0.048 (21 days of mean recovery time considering both mild and severe cases).

## Results and Discussion

The above delay differential equation is used for all the states in the United States, and we found 3 groups among the states according to the accuracy of the data. The active cases reported on Worldometer^1^ for a few states show excellent agreement with our estimation of active cases. One example state for this group is Texas, as seen in Figure 1(a). There are few states in the second group that are largely not matching with the analytical estimation, indicating that reported active data are inaccurate. These states are listed in column 2 in Table 1 and 1 representative state is Virginia as seen in Figure 1(b), where the current active cases are reported to be 530 820 which should have been just around 31 237, according to our calculation. Interestingly, in the last group, there are some states for which the reported active cases follow the estimated active cases for some days; however, the trend of the curve changes and does not follow our estimation as represented in Indiana, shown in Figure 1(c). In Figure 1, the reported total and active cases with the estimated active cases for one of the states in all the 3 groups are shown, and the figures for the remaining states are given in the Supplementary Material.

**Figure 1.**
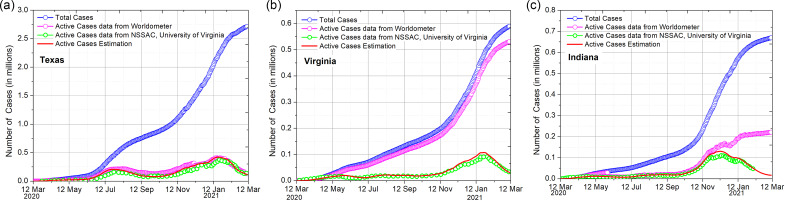
**(a)** Texas representing group states in which active data are reported correctly. **(b)** Virginia represents the second group in which data are largely incorrect. **(c)** Indiana represents the third group in which data are partially correct. The NSSAC, University of Virginia, data for the active cases are in close agreement with our analytical estimation.

The Network Systems Science and Advanced Computing (NSSAC) division of the Biocomplexity Institute and Initiative at the University of Virginia has created a visualization tool that presents a way of examining data curated by different data sources.^10^ We compared the active cases data provided by NSSAC and found that this independent source of active data is in close agreement with the corrected active cases data. The recovery rate in individual states may vary to an extent of ±10% depending on the variation in the number of tests performed, fraction of mild and severe cases. However, we have taken a uniform value of the recovery rate as 0.048 for all the US states. The mortality rate can be calculated from the active cases data,^2^ as shown:
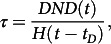



where, 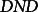 are the daily new extinct cases, and 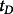 is the delay associate with the extinct cases as explained in the mortality rate calculation.^2^ In the initial phases of infection, many of the states show higher active cases than the true values that give a mortality rate lower than the actual rate. Since the hospital bed estimation, intensive care unit equipment requirement estimation depends on the active cases currently and that are expected in the near future, and correction in the data facilitates better management of these entities. For the purpose of modeling and prediction, it is important that a mathematical model is validated against the data. Correct active cases data imply the right model parameters and a more accurate estimation of the hospital requirements.

## Conclusion

The reported active cases for a few states are consistent with the total detected cases and death cases for a recovery rate parameter value of 0.048. For a few states, the data have been corrected recently. However, for 9 states, the active cases data are still largely incorrect. We generate the corrected active case data for all states, report them in the Supplementary Material, and also keep the data available on GitHub.

## Data Availability

The data for the corrected active cases for all the US states can be downloaded using the GitHub link: https://github.com/ravisolankigithub/covid-activecases-usa.git.

## Supplementary material

For supplementary material accompanying this paper visit https://doi.org/10.1017/dmp.2021.130. click here to view supplementary material
