# Supplementary material for: Correction in Active Cases Data of COVID-19 for the US States by Analytical Study
Source: Disaster Med Public Health Prep. 2021 Apr 30:1–4. doi: 10.1017/dmp.2021.130 (PMC8267334; doi:10.1017/dmp.2021.130)
Supplement: Supplementary file 1 [file S1935789321001300sup001.pdf]

SUPPLEMENTARY MATERIAL

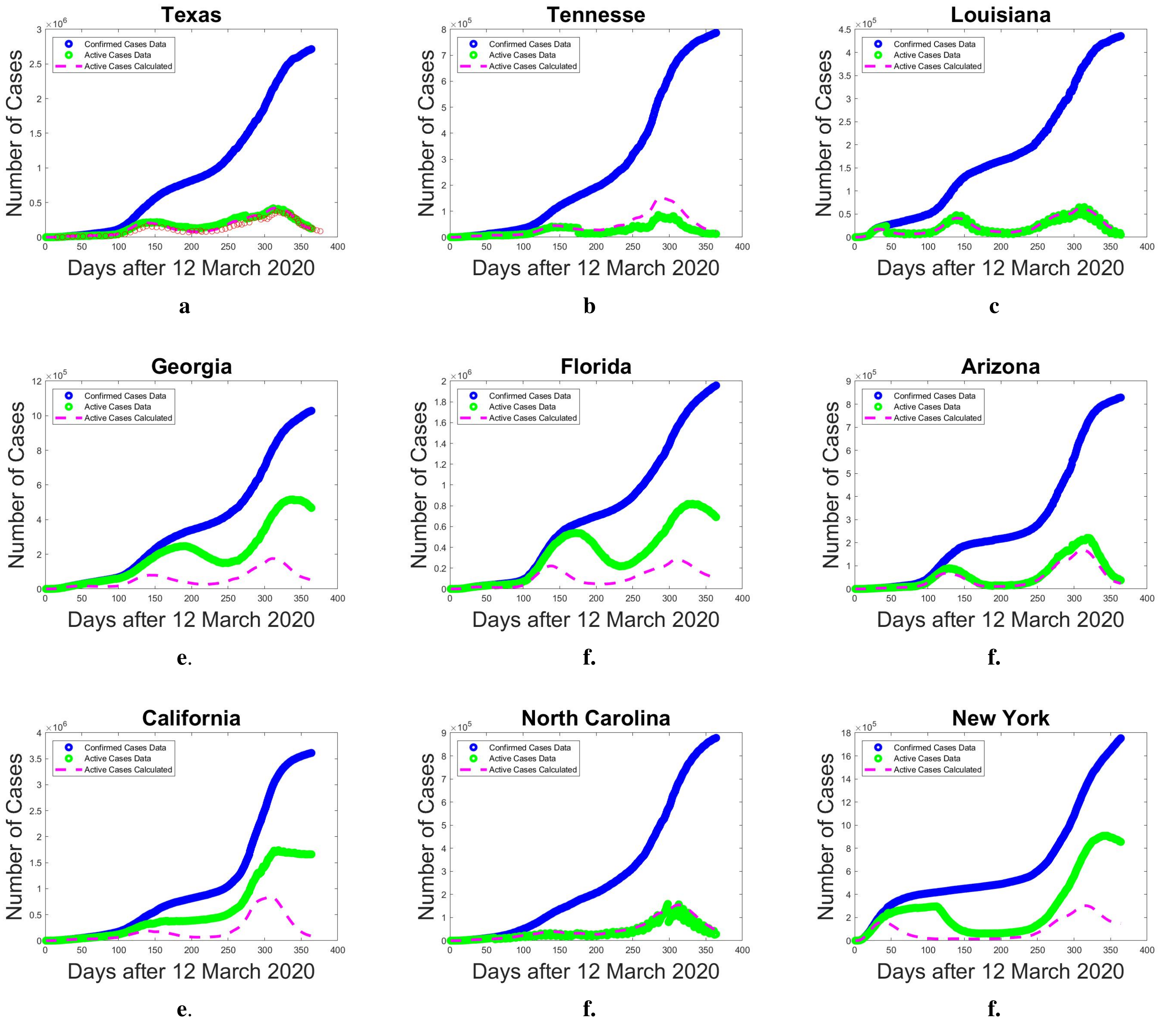

Fig. **S1**: Reported total cases, active Cases data and analytically estimated active cases for various US states

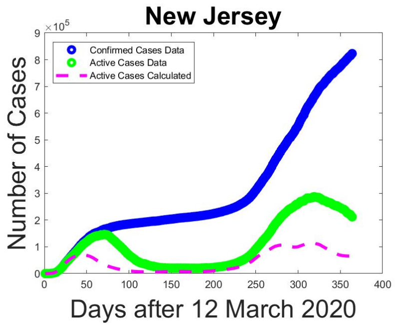

a

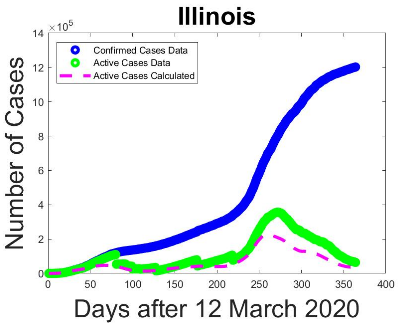

b

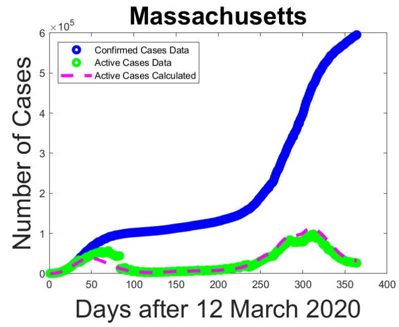

c

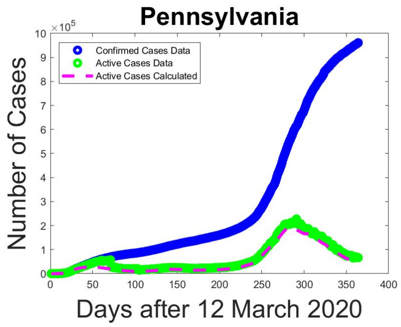

e.

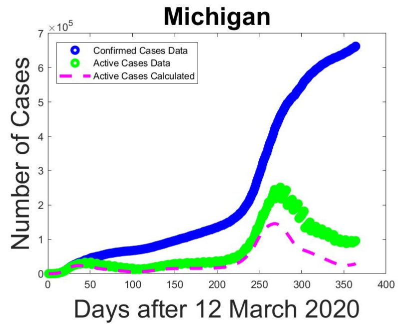

f.

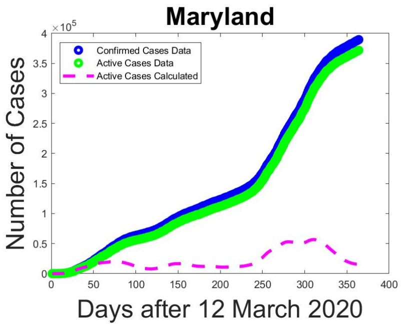

f.

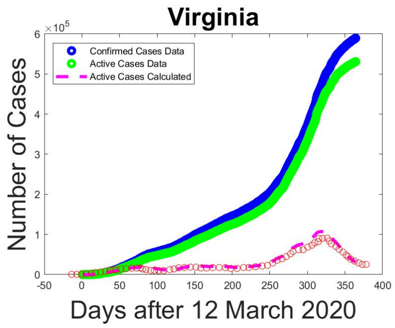

e.

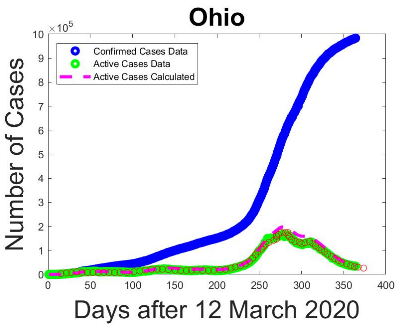

f.

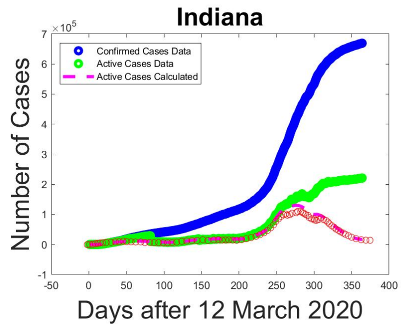

f.

Fig. **S2**: Reported total cases, active Cases data and analytically estimated active cases for various US states

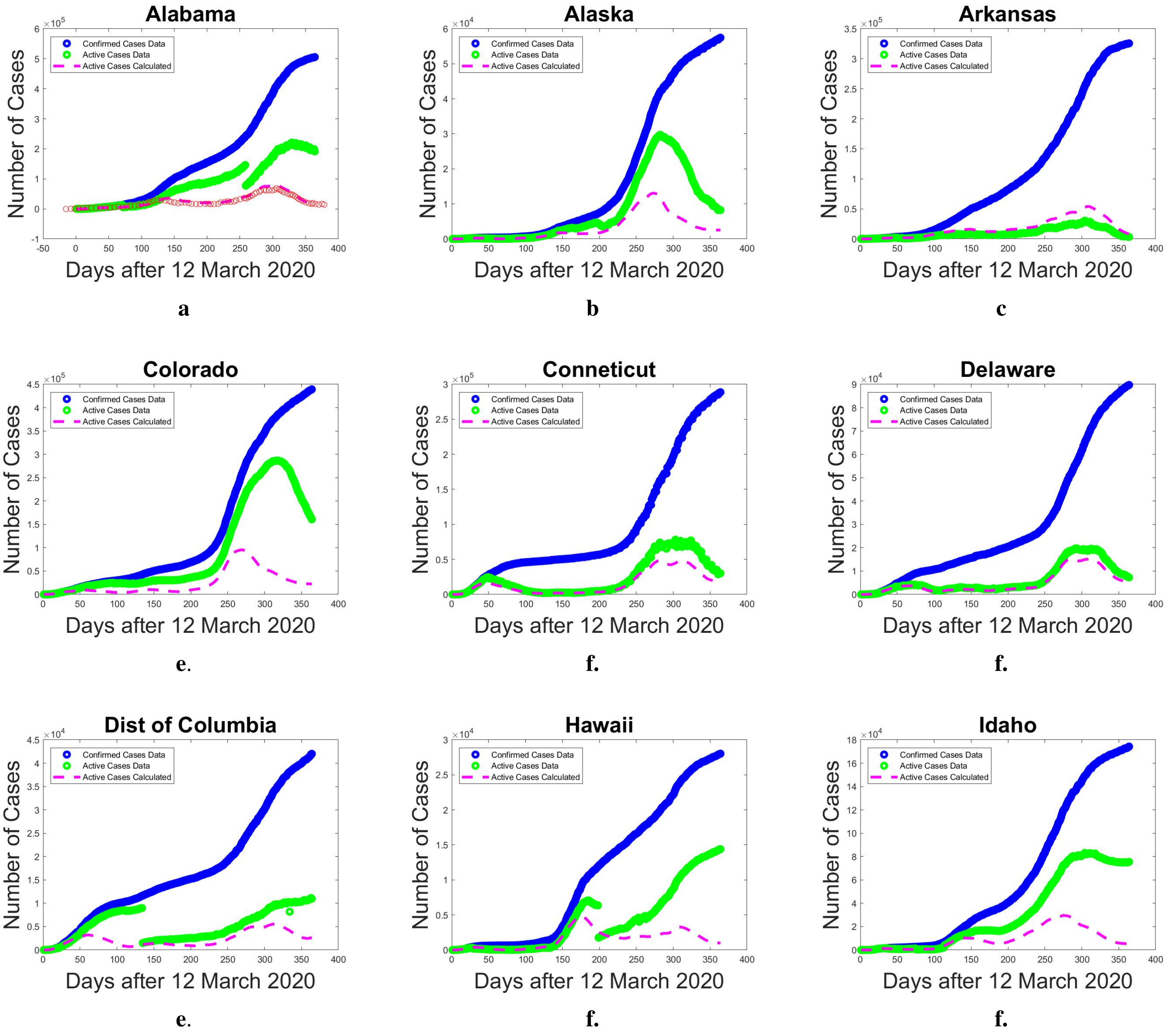

Fig. **S3**: Reported total cases, active Cases data and analytically estimated active cases for various US states

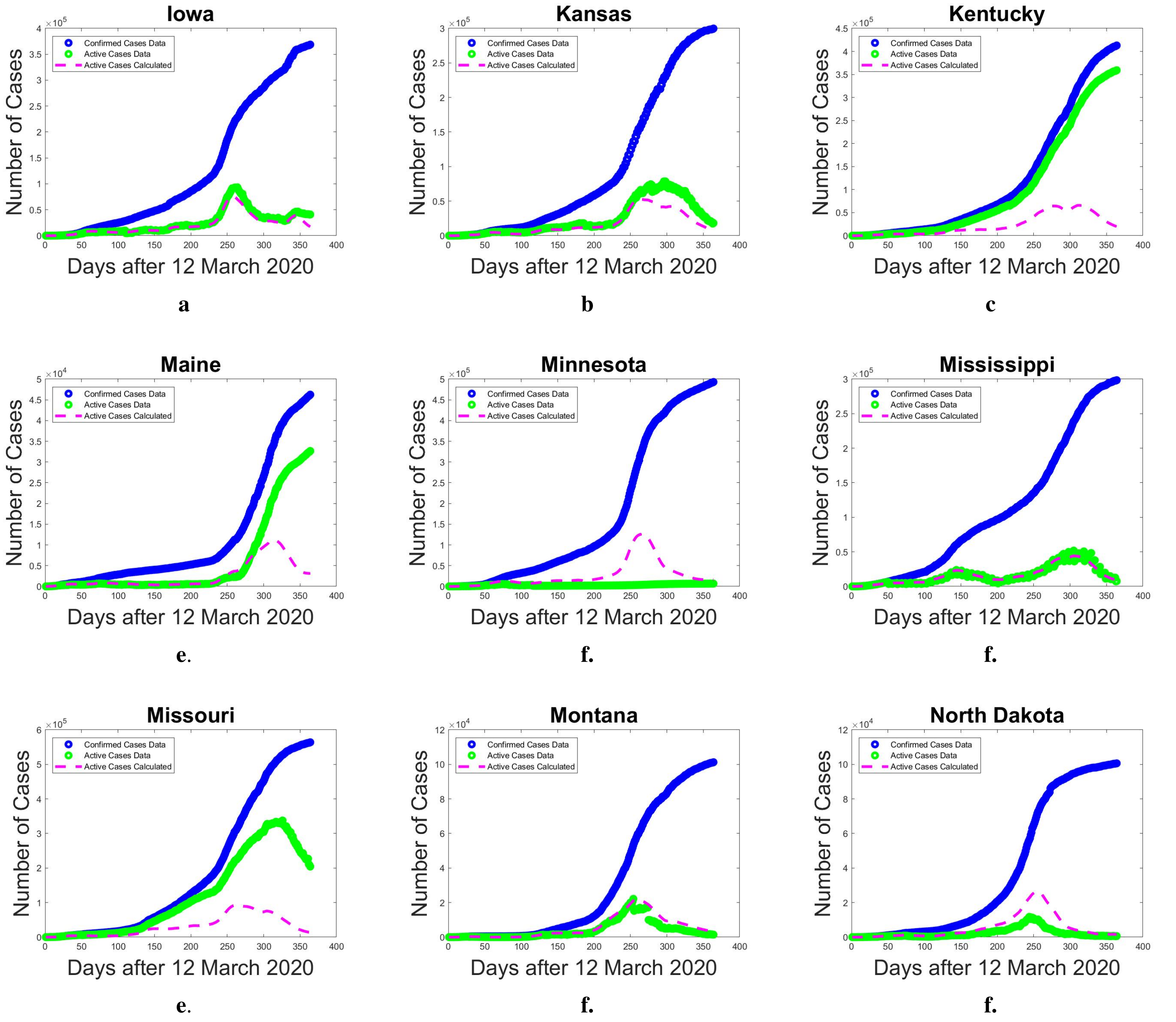

Fig. **S4**: Reported total cases, active Cases data and analytically estimated active cases for various US states

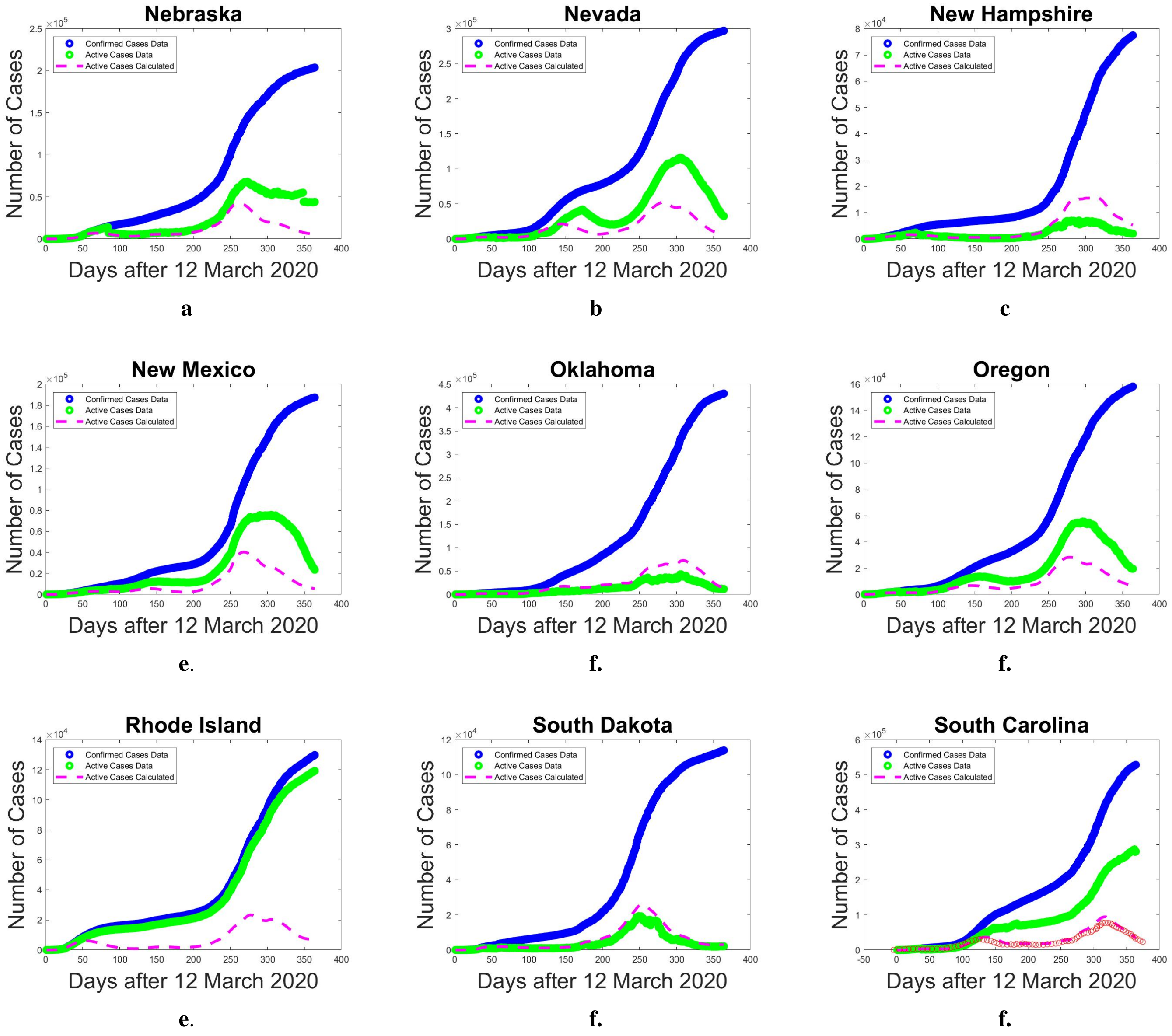

Fig. **S5**: Reported total cases, active Cases data and analytically estimated active cases for various US states

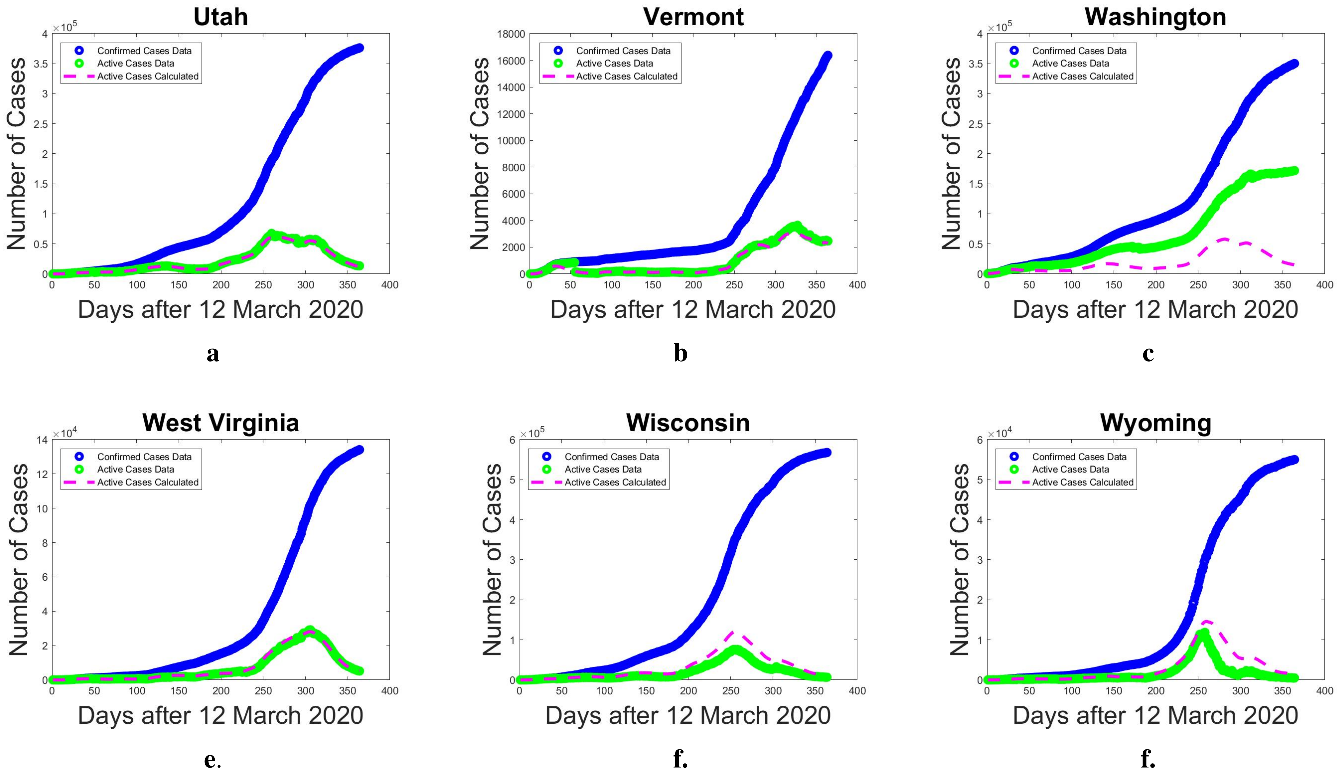

Fig. **S6**: Reported total cases, active Cases data and analytically estimated active cases for various US states
